# Impact and influence of crystallography across the sciences

**DOI:** 10.1107/S2052252516017012

**Published:** 2016-10-27

**Authors:** Samar Hasnain

**Affiliations:** a Max Perutz Professor of Molecular Biophysics at the University of Liverpool and Editor-in-Chief of IUCr Journals, Barkla X-ray Laboratory of Biophysics, Institute of Integrative Biology, Life Sciences Building, Liverpool, L69 7ZB, United Kingdom

**Keywords:** crystal engineering, two-dimensional materials, serial crystallography, cryo-EM, personalized medicine, graphene, molecular machines, enzymes

## Abstract

Crystallography has influenced many of the traditional science disciplines and has opened a number of cross-disciplinary activities often bringing physicists, chemists, biologists and medical scientists together.

How does one assess the impact of a discipline, particularly one that spans across the sciences and requires a cultural change in the way of thinking and looking at a problem? Crystallography, whether using electrons, neutrons or X-rays, has transformed the way we look at a problem and the level at which we wish to glean the composition of a material and its internal arrangement. Whether it is something as simple as table salt or as complex as the ribosome, crystallography has provided an insight that no other approach could provide. The first diffraction experiment on a single crystal of copper sulfate by Max von Laue in 1912 (Friedrich *et al.*, 1912 ▸) and subsequent interpretation by Lawrence Bragg (Bragg, 1913 ▸) gave birth to the field.

In 1912, James Batcheller Sumner joined Harvard Medical School in order to study biochemistry with Professor Otto Folin. Sumner, who had lost an arm due to a firearm accident during a hunting outing with a friend, was advised by Folin to take up law, since he thought that a one-armed man could never make a success of chemistry. Sumner obtained his PhD degree in biochemistry in 1914, the year that Laue received his Nobel Prize, and became an Assistant Professor of Biochemistry at Cornell Medical School, Ithaca, New York, where he became a full Professor of Biochemistry in 1929. During this time, he continued to extract an enzyme, urease, in pure form through crystallization (this work was carried out against serious reservations, with many regarding Sumner’s idea of isolating urease as ridiculous). In 1926, he succeeded in crystallizing the first enzyme with high activity (Sumner, 1926 ▸), but most biochemists remained sceptical. This accomplishment provided incontrovertible proof that enzymes were well-defined chemical compounds thus playing a historical role in the development of enzymology. The method began to gain a wider acceptance as a general purification method for enzymes, when John Howard Northrop obtained crystalline pepsin, trypsin and chymotrypsin enzymes, which are active in the digestive process. In 1935, Wendell Meredith Stanley was able to extract the tobacco mosaic virus, from considerable quantities of infected tobacco leaves, in the form of pure crystals. They shared the Nobel Prize in Chemistry 1946; one half was awarded to James Batcheller Sumner *‘for his discovery that enzymes can be crystallized’*, the other half jointly to John Howard Northrop and Wendell Meredith Stanley *‘for their preparation of enzymes and virus proteins in a pure form’*. The impact of this work on modern biology cannot be overemphasized. Sumner’s approach was transformative receiving nominations for the Nobel Prize in Physiology and Medicine as well as in Chemistry. He received his first nomination in 1932. Sumner was elected to the National Academy of Sciences (USA) in 1948, the year in which the IUCr started its first journal, *Acta Crystallographica*, which aimed to provide a home for all aspects of crystallographic research. It is worth noting that the structure of jack bean urease was determined only in 2010 (Balasubramanin & Ponnuraj, 2010 ▸), with the structure of a bacterial urease in 1995 (Jabri *et al.*, 1995 ▸).

The next giant step was to come in 1962 and 1964 when three Nobel Prizes were awarded, two in Chemistry [to Kendrew and Perutz (1962) *‘for their studies of the structures of globular proteins’* (Dickerson *et al.*, 1961 ▸; Kendrew *et al.*, 1960 ▸; Perutz, 1956 ▸; Perutz *et al.*, 1960 ▸) and to Dorothy Hodgkin (1964) *‘for the structure of many biochemical substances including Vitamin B12’* (Brink *et al.*, 1954 ▸)] and one in Physiology and Medicine to Francis Crick, James Watson and Maurice Wilkins *‘for establishing the helical structure of DNA’.* The impact of these breakthroughs on biology, chemistry and medicine is immeasurable given the opportunities offered by genomic science, which is promising to make personalized medicine feasible in many parts of the world.

In the current decade, the ultimate recognition of a Nobel Prize has been acheived across a whole range of sciences, demonstrating the outreach of crystallography. The 2013, 2012 and 2011 Chemistry awards went to Martin Karplus and Michael Levitt *‘for the development of multiscale models for complex chemical systems’,* to Robert Lefkowitz and Brian Kobilka *‘for studies of G-protein-coupled receptors’*, and to Danny Shechtman *‘for the discovery of quasicrystals’,* respectively. In 2010, Andre Geim and Kostya Novoselov received the Physics Nobel Prize*‘for groundbreaking experiments regarding the two-dimensional material graphene’*, opening a whole new field of two-dimensional materials chemistry. We congratulate the 2016 Nobel Prize winners in Physiology and Medicine (Yoshinori Ohsumi *‘for his discoveries of mechanisms for autophagy’*) and Chemistry (Jean-Pierre Sauvage, Sir J. Fraser Stoddart and Bernard L. Feringa *‘for the design and synthesis of molecular machines’*). We are pleased to note that Ohsumi, Stoddart and Feringa have published some 40 papers in IUCr journals during the period 1986–2014.

One hundred years after the first Nobel Prize to crystallography to Laue in 1914, the IUCr celebrated the impact and influence of crystallography by launching a comprehensive, all inclusive open-access journal, **IUCrJ**. The journal received its first impact factor of 5.3 this year. This starting impact factor is pleasing and results from our authors having the trust and confidence to submit some of their best work to the journal. We encourage you all to consider the journal alongside other notable journals such as *PNAS*, *JACS*, *Nature Communications* and *Nature Materials*. The top ten countries contributing to the journal were the USA, the UK, Germany, France, India, Japan, Denmark, Switzerland, Australia and China/Russia/Spain; eight of these are common with *Nature*, *Science* and *PNAS*. The five top contributing countries of the 30 most cited papers (with a total of 602 citations and averaging 20 cites per paper) were Germany, the USA, the UK, France, India and Switzerland.

These highly cited papers represent the full range of science, methods and instrumentation. The broad area of science covered was from halogen bonds (Mukherjee & Desiraju, 2014 ▸) and multiferroic materials (Gilioli & Ehm, 2014 ▸) to serial femtosecond crystallography (Gati *et al.*, 2014 ▸; Schlichting, 2015 ▸) and synchrotron radiation serial crystallography (Stellato *et al.*, 2014 ▸). In addition to reporting step changes in science, some of these articles reported major advances in instrumentation and approaches. The journal thus provides readers with an opportunity to see some excellent science in the chemical, materials and biological fields while keeping up with significant advances in instrumentation, methods and approaches. We aim to continue this unique combination of structural sciences in one place while welcoming new areas like the chemistry and materials science pertaining to two-dimensional crystals such as graphene (Novoselov *et al.*, 2005 ▸).

Like the FEL community, we wish to encourage the cryo-EM community to make **IUCrJ** their natural home. The importance of cryo-EM for structural science has been obvious to the IUCr for many years and will be an important feature of the next IUCr Congress in Hyderabad (http://www.iucr2017.org/), where as well as the keynote speakers of the IUCr Gjonnes Medal (Richard Henderson and Nigel Unwin), there will be an additional keynote (Sriram Subramanian) and three microsymposia each with six talks. Like the Congress, **IUCrJ** aims to be a leading journal for reporting important advances in cryo-EM methods as well as significant science results from the application of cryo-EM, which is proving to be the method of the decade.

We are pleased to announce that the IUCr has signed the San Francisco Declaration on Research Assessment (DORA, http://www.ascb.org/dora/). The Declaration calls on the world scientific community to stop using journal-based metrics, particularly journal impact factors, as a surrogate measure of the quality of individual research articles, or in the assessment of individual scientists’ contributions. It is clear from a citation analysis of *PNAS*, **IUCrJ** and *Nature* that a large proportion of papers in these journals receive much lower numbers of citations than the journal’s impact factor, making use of such metrics for hiring, promotion or funding decisions irrational. The policy of IUCr Journals is to present impact factors within the context of a broader range of journal-based metrics such as five-year impact factors, Eigenfactors, publication times *etc*. We also publish journal citation distributions for each of our journals. We emphasize that even these extended metrics are merely indicators of the performance of a journal and must not be used for assessing an individual scientist’s performance for recruitment, promotion, or funding decisions.
